# Inescapable foot shock induces a PTSD-like phenotype and negatively impacts adult murine bone

**DOI:** 10.1242/dmm.050044

**Published:** 2024-01-12

**Authors:** Sara J. Sidles, Ryan R. Kelly, Kirsten D. Kelly, Jessica D. Hathaway-Schrader, Stephanie K. Khoo, Jeffrey A. Jones, James J. Cray, Amanda C. LaRue

**Affiliations:** ^1^ Research Service, Ralph H. Johnson Department of Veterans Affairs Health Care System, Charleston, SC 29401, USA; ^2^ Department of Pathology and Laboratory Medicine, Medical University of South Carolina, Charleston, SC 29425, USA; ^3^ College of Dental Medicine, Medical University of South Carolina, Charleston, SC 29425, USA; ^4^ Department of Surgery, Medical University of South Carolina, Charleston, SC 29425, USA; ^5^ Division of Anatomy, The Ohio State University, Columbus, OH 43210, USA

**Keywords:** Post-traumatic stress disorder, Animal model, Inescapable foot shock, Bone, Osteoporosis, Inflammation

## Abstract

Post-traumatic stress disorder (PTSD) is associated with osteopenia, osteoporosis and increased fracture risk in the clinical population. Yet, the development of preclinical models to study PTSD-induced bone loss remains limited. In this study, we present a previously unreported model of PTSD in adult female C57BL/6 mice, by employing inescapable foot shock and social isolation, that demonstrates high face and construct validity. A subset of mice exposed to this paradigm (i.e. PTSD mice) display long–term alterations in behavioral and inflammatory indices. Using three–dimensional morphometric calculations, cyclic reference point indentation (cRPI) testing and histological analyses, we find that PTSD mice exhibit loss of trabecular bone, altered bone material quality, and aberrant changes in bone tissue architecture and cellular activity. This adult murine model of PTSD exhibits clinically relevant changes in bone physiology and provides a valuable tool for investigating the cellular and molecular mechanisms underlying PTSD-induced bone loss.

## Teaser

**Summary:** A murine model of post-traumatic stress disorder (PTSD)-induced bone loss serves as a valuable tool to investigate the underlying mechanisms of osteoporosis associated with PTSD.

## INTRODUCTION

Post-traumatic stress disorder (PTSD) is a mental health condition that may develop after experiencing or witnessing a life-threatening or traumatic event. PTSD impacts 7-8% of individuals in the USA annually. Some populations show an increased incidence of PTSD, including veterans of the armed forces, for whom rates have approached 20% in those returning from recent conflicts in Afghanistan and Iraq, and 30% for Vietnam veterans (Kelly et al., 2019). Clinically, PTSD is defined by a cadre of symptoms that can persist for months to years, including a heightened response to events related to the initial traumatic event (i.e. the ‘index event’). The Diagnostic and Statistical Manual of Mental Disorders (DSM)-5 describes eight major symptom criteria for PTSD, including an initiating stressor (A), intrusive re-living of the initial event (B), avoidance (C), negative changes in mood or cognition (D), changes in arousal or reactivity (E), and a persistent display of symptoms (F) that significantly affect functioning (G) and cannot be attributed to other causes (H) (Shalev et al., 2017). Together, these symptoms have a profound impact on quality of life (QOL) and can have severe anxious, depressive and debilitating effects, as well as enhancing vulnerability to suicide and substance or alcohol abuse (Schafer and Najavits, 2007; Brady et al., 2000; Gradus et al., 2017).

Recent evidence suggests that PTSD is associated with increased risk for other psychiatric disorders, autoimmune disorders, sleep apnea, cardiovascular disease, obesity, diabetes, lung pathologies and musculoskeletal disorders (Kelly et al., 2019). Of significant concern, PTSD has been linked to reductions in bone density and bone mass, as well as to increased fracture risk (Azuma et al., 2015; Huang et al., 2018; Neigh and Ali, 2016). Clinical studies show that US Veterans diagnosed with PTSD have a higher risk of developing osteoporosis, which accounts for >1.5 million fractures annually in the total US Veteran population (El-Gabalawy et al., 2018; Kelly et al., 2019; Hain et al., 2011). By 2025, osteoporosis-related treatment costs are estimated to exceed $25 billion (Kelly et al., 2019). Further complicating the risk for bone comorbidity, the standard pharmacological treatment for PTSD often includes prescription of selective serotonin reuptake inhibitors (SSRIs), which can negatively impact bone health in the long term (Wadhwa et al., 2017; Williams et al., 2018). Over a 10-year period, PTSD patients prescribed SSRIs were 56.7% more likely to suffer a hip fracture and 34.6% more likely to develop osteoporosis compared with SSRI-naïve PTSD patients (Brinton et al., 2019). Thus, understanding the impact of PTSD on bone is crucial. Despite recent identification of comorbidities of PTSD, there remains a lack of understanding of the physiological and biochemical mechanisms driving PTSD-associated bone pathology.

Several studies have indicated a potential link between stress and bone pathophysiology. Using a chronic psychosocial stress model in adolescent mice, Foertsch et al. demonstrated reduced tibia and femur lengths, increased growth-plate thickness, and disruptions in endochondral ossification that led to altered skeletal development (Foertsch et al., 2017). Stress hormone signaling via the brain-immune axis has been implicated as a driver in disrupting the osteoblast:osteoclast balance (Bottaccioli et al., 2019; Kelly et al., 2020). In a mouse model of psychosocial stress with induced fracture, reduced growth plate endochondral ossification was accompanied by altered immune cell infiltration at the fracture site and increased expression of tyrosine hydroxylase, a catalytic enzyme involved in catecholamine biosynthesis, by growth plate-residing bone marrow cells (Haffner-Luntzer et al., 2019). Inflammatory mediators, including interleukin (IL) 17A and tumor necrosis factor (TNF) α, have been shown to directly promote osteoclast differentiation (Weitzmann, 2017; Pietschmann et al., 2016), while inflammatory cells, including activated T cells, have been implicated in the pathophysiology of postmenopausal osteoporosis (Weitzmann and Ofotokun, 2016). Several immune mediators that drive osteoclastogenesis in favor of net bone loss, including interferon (IFN) γ, IL6, TNFα, and IL17A, have also been implicated in the development of PTSD and comorbid conditions (Neigh and Ali, 2016; Passos et al., 2015; Wang et al., 2017). These and other studies indicate that both systemic (hematopoiesis and immune mediators) and local (immune cell infiltration) physiological changes may play a role in disrupting the osteoblast:osteoclast balance, ultimately leading to bone loss (Chrousos, 2000; Dhabhar, 2014; Neigh and Ali, 2016; Heidt et al., 2014).

The primary goal of the current study was to develop a clinically relevant adult murine model of PTSD that exhibits changes in bone. Such a preclinical model would allow in-depth study of the mechanisms driving PTSD-induced bone loss. Modeling PTSD in small animals poses a significant challenge, as it is a highly heterogeneous disorder (Blevins et al., 2015). Animal models of PTSD rely on face validity of main DSM-5 symptom clusters and are interpreted in relation to the produced PTSD-related phenotypes (Deslauriers et al., 2018; Verbitsky et al., 2020). Inescapable foot shock (IFS), including the fear conditioning and extinction paradigm, is highly relevant to PTSD, and has been shown to evoke PTSD-like constructs, as well as biological and physiological phenotypes associated with PTSD (Deslauriers et al., 2018; Flandreau and Toth, 2018). Variations of the IFS model also elicit changes in fear circuitry, including changes in neural activation (prefrontal cortex/amygdala), morphology (hippocampus) and signaling [hypothalamus-pituitary axis (HPA)], supporting the construct and predictive validity of this paradigm (Deslauriers et al., 2018; Flandreau and Toth, 2018; Aspesi and Pinna, 2019; Mahan and Ressler, 2012).

The murine model of IFS presented herein is based on social isolation paired with an IFS paradigm in which a series of unavoidable foot shocks are delivered as the trauma-inducing index event. Through a battery of behavioral tests, including trauma reminder, locomotor and sociability tests, we show that this model induces a PTSD-like phenotype in adult female B6.SJL-Ptprc^a^Pepc^b^/BoyJ (C57BL/6) mice, and elicits trauma-specific freezing, social avoidance and hyperactivity that persists for at least 8 weeks after IFS index trauma in a subset of mice (i.e. PTSD mice). Further, we show that several markers of inflammation, including IFNγ, IL6 and TNFα, are elevated systemically in PTSD mice 15 weeks post-IFS index trauma, supporting the etiological validity of this model. Trabecular bone loss and architectural changes were observed in PTSD mice relative to control mice, based on micro-computed tomography (micro-CT), cyclic reference point indentation (cRPI) calculations and femur histomorphometric analyses. Using composite scoring across behavioral and bone assessments, data indicate an inverse relationship between PTSD scores and trabecular bone health scores, thus pointing to this IFS model of PTSD as a useful tool for understanding the mechanistic drivers of PTSD-induced bone pathology.

## RESULTS

### Inescapable foot shock induces trauma-associated freezing behavior in female C57BL/6 mice

**Fig. 1.**
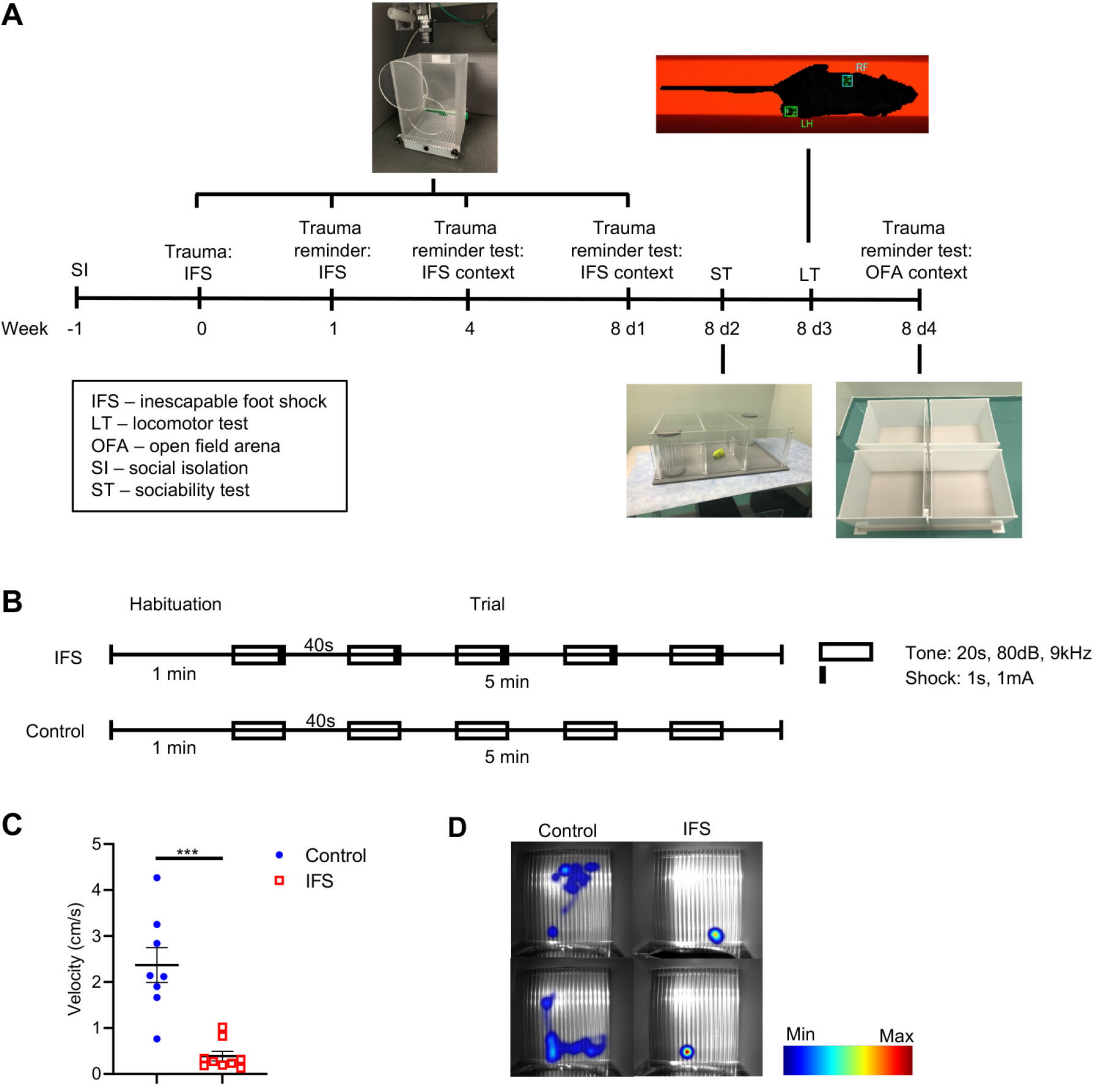
**PTSD paradigm in female C57BL/6 mice.** (A) The inescapable foot shock (IFS)-based model of PTSD in female C57BL/6 mice employs social isolation 1 week before IFS index trauma, followed by a series of trauma reminder tests over an 8-week period. Eight weeks post-IFS trauma, a battery of behavioral tests, including a trauma reminder test in the IFS context, a sociability test (ST), a locomotor test (LT) and a trauma reminder test in open field arena (OFA) context, are conducted to assess the PTSD-like phenotype. (B) IFS index trauma paradigm on day 0, in which IFS mice were placed singly into inescapable arenas and allowed to habituate for 1 min before delivery of five 1 s, 1 mA foot shocks over a 5 min trial. Each shock was preceded by and co-terminated with a 20 s, 80 dB, 9 kHz tone cue. Control mice were placed singly into the arenas and allowed to habituate for 1 min before delivery of five 20 s, 80 dB, 9 kHz tone cues (no shocks) over a 5 min trial. Velocity (cm/s) of mice was recorded with an infrared-camera at a rate of 25 frames/s and EthoVision XT v13 software. (C) Velocity (cm/s) of mice in IFS arenas after the fifth tone (control) or tone-shock (IFS) event during IFS trauma paradigm on day 0. (D) Heat map depicting locomotion of representative control and IFS mice in IFS arenas after the fifth tone (control) or tone-shock (IFS) event during IFS trauma paradigm on day 0. Data are mean±s.e.m. Control, *n*=8; IFS, *n*=9. In IFS, two mice are omitted from C due to incomplete camera tracking. Mann–Whitney *U*-test (***P=0.0003).

As the fear conditioning and extinction paradigm is highly relevant to PTSD and evokes PTSD–like constructs (Flandreau and Toth, 2018; Deslauriers et al., 2018), the current model was based on social isolation paired with inescapable foot shock (IFS) as the index traumatic event in adult female C57BL/6 mice (Fig. 1A). One week before IFS index trauma, mice in the experimental group (IFS, *n*=11) were singly housed (social isolation) and remained singly housed for the duration of the study. On day 0, IFS mice were singly placed into inescapable arenas within sound-attenuated chambers and allowed to habituate for 1 min before delivery of five, 1 s, 1 mA foot shocks over a 5 min trial (Fig. 1B, solid bars). Each shock was preceded by and co-terminated with a single 20 s, 80 dB, 9 kHz tone cue. Control mice (*n*=8) were placed singly into arenas and allowed to habituate for 1 min before delivery of five 20 s, 80 dB, 9 kHz tone cues over a 5 min trial. Control mice did not receive any foot shocks over the trial. Freezing behavior after tone-shock/tone delivery was recorded using an infrared (IR) camera at 25 frames/s and EthoVision XT v13 software (Noldus). After the fifth tone-shock delivery, IFS mice exhibited decreased mean velocity (cm/s) compared with control mice (–83.5%, *P*=0.0003), demonstrating freezing behavior in response to the trauma-specific cue (Fig. 1C,D).

### Subset of female C57BL/6 mice undergoing the IFS paradigm exhibit PTSD-like behaviors 8 weeks post-IFS index trauma

**Fig. 2.**
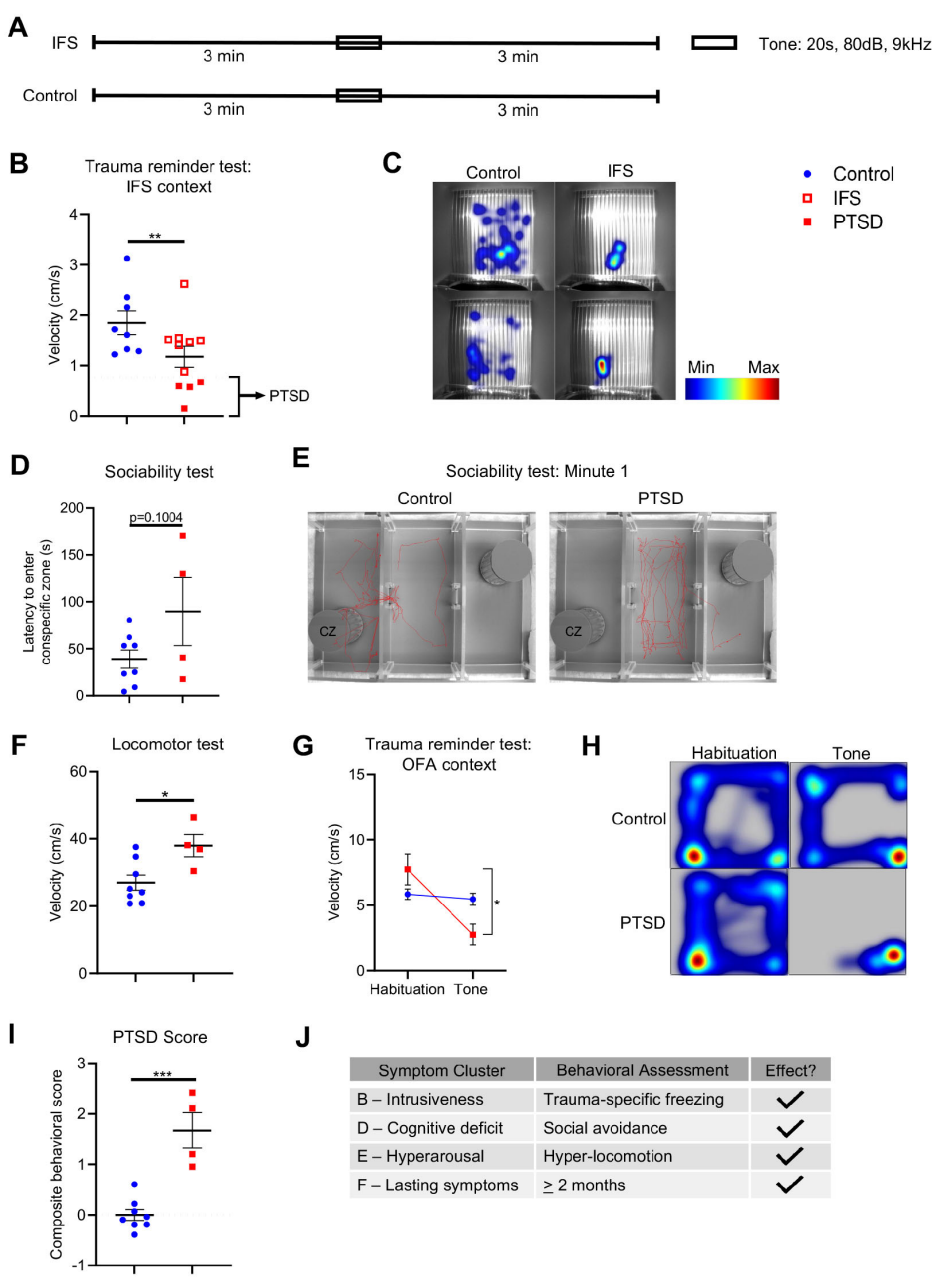
**A subset of mice undergoing IFS trauma exhibit PTSD-like behaviors 8 weeks post-IFS index trauma.** (A) Eight weeks after IFS index trauma, control and PTSD mice were singly placed into IFS arenas and allowed to habituate for 3 min before delivery of a single 20 s, 80 dB, 9 kHz tone (trauma reminder test in IFS context). Velocity (cm/s) of mice was recorded with an infrared camera at a rate of 25 frames/s and EthoVision XT v13 software. (B) A subset of mice in the IFS group (i.e. PTSD mice) exhibited decreased velocity (cm/s) after the tone event in the trauma reminder test. (C) Heat map depicting locomotion of representative control and IFS mice in IFS arenas after the tone event in the IFS trauma reminder test in IFS context. (D) Eight weeks after IFS index trauma, control and PTSD mice were placed singly into a three-chambered sociability arena and allowed to explore freely, with an age-matched, sex-matched conspecific subject placed in a plexiglass cage in one chamber (sociability test). Overall locomotion and latency to enter the zone directly surrounding the conspecific subject (s) was recorded for each mouse using an infrared camera at 25 frames/s and EthoVision XT v13 software. PTSD mice exhibited increased latency (s) to enter the zone directly surrounding the conspecific subject. (E) Track map depicting locomotion of representative control and PTSD mice in the three-chambered sociability arena during the first minute of the sociability test (CZ, conspecific zone). (F) Eight weeks after IFS index trauma, control and PTSD mice were placed singly onto an enclosed, illuminated glass platform and allowed to cross freely to a goal box for a total of three compliant runs/mouse (locomotor test). Body velocity (cm/s) was captured by an undermounted, high-speed color camera and CatWalk XT software. PTSD mice exhibited increased average velocity (cm/s) across the platform compared with control mice. (G) Eight weeks after IFS index trauma, control and PTSD mice were placed singly into open field arenas (OFA) and allowed to habituate for 3 min before delivery of a single 20 s, 80 dB, 9 kHz tone (trauma reminder test in OFA context). Velocity (cm/s) of mice was recorded with an infrared camera at a rate of 25 frames/s and EthoVision XT v13 software. PTSD mice exhibited decreased velocity (cm/s) during the tone event compared with habituation (*P*=0.0140), whereas there was no change in control velocity between habituation and tone (*P*=0.2819). (H) Heat map depicting locomotion of control and PTSD mice in OFA arenas during the tone event in the IFS trauma reminder test in OFA context. (I) An average composite score across all behavioral assessments eight weeks post-IFS index trauma (PTSD score) was calculated for control and PTSD mice. PTSD mice exhibited a higher PTSD score compared with control mice. (J) PTSD mice exhibit PTSD-like behaviors in behavioral assessment performed 8 weeks after IFS index trauma. Data are mean±s.e.m. Control, *n*=8; PTSD, *n*=4. Unpaired two-tailed Student's *t*-test (*P<0.05, **P<0.01, ***P<0.001).

Eight weeks after IFS index trauma, PTSD-like behaviors were evaluated in IFS and control mice through a series of behavioral assessments, including a trauma reminder test in the IFS context, sociability test, locomotor test and trauma reminder test in open field arena (OFA) context (Fig. 1A). To evaluate trauma-specific freezing behavior in the IFS context, IFS and control mice were placed singly into IFS arenas and allowed to habituate for 3 min before delivery of a single 20 sec, 80 dB, 9 kHz tone cue (Fig. 2A). Subject velocity (cm/s) and overall locomotion was recorded using an IR camera at 25 frames/s and EthoVision XT v13 software. Compared with control mice, a subset of the 11 IFS mice (*n*=4; termed PTSD mice) exhibited persistent, decreased mean velocity (–73.1%, *P*=0.003) for 3 min after tone delivery (Fig. 2B,C). PTSD mice were defined based on the following criterion: velocity_PTSD_ ≥1.5 standard deviations below mean velocity_Control_. To evaluate social avoidance, a three-chambered sociability test was employed. Subjects were introduced singly to a three-chambered arena and allowed to explore freely for 5 min (habituation) before an age- and sex-matched conspecific subject was placed within a plexiglass-barred cage in one of the chambers. The test subject was then reintroduced to the arena and allowed to explore freely for 5 min (test). Time spent exploring each chamber and latency to enter the zone directly surrounding the conspecific was recorded using an overhead camera at 25 frames/s and EthoVision XT v13 software. Compared with control mice, PTSD mice exhibited a trend toward increased latency or latencies to enter the conspecific zone (+130.8%, *P*=0.1004, Fig. 2D). Within the first minute of the sociability test, 75% of control mice entered the conspecific zone, compared with 50% of PTSD mice (Fig. 2D,E). To assess hyperactivity, subjects were singly placed onto an enclosed, illuminated glass platform (CatWalk XT) and allowed to traverse freely to a dark goal box for a total of three compliant runs (locomotor test). Body velocity (cm/s) was captured by an under-mounted, high-speed color camera. Compared with control mice, PTSD mice exhibited significant hyper-locomotion, defined by increased mean body velocity (+40.9%, *P*=0.0188, Fig. 2F). To evaluate trauma-specific freezing in a novel environment, mice were placed singly into open field arenas and allowed to habituate for 3 min before delivery of a single 20 sec, 80 dB, 9 kHz tone cue. Subject velocity (cm/s) and overall locomotion was recorded using an IR camera at 25 frames/s and EthoVision XT v13 software. PTSD mice exhibited significantly decreased velocity (cm/s) during tone delivery compared with habituation (-64.3%, *P*=0.0140, Fig. 2G,H), demonstrating trauma-specific freezing in a novel context at 8 weeks post-IFS index trauma. Control mice exhibited no change in velocity in response to tone delivery in a novel environment (*P*=0.2819, Fig. 2G,H). To evaluate overall PTSD-like phenotype, a composite score (i.e. PTSD score) was calculated for each subject based on behavioral assessments (i.e. trauma reminder tests in IFS and OFA contexts, sociability test, and locomotor test). PTSD mice exhibited a significantly higher PTSD score compared with control mice (*P*=0.0002, Fig. 2I). Together, these data demonstrate that the IFS trauma and social isolation paradigm elicits a PTSD–like phenotype, representing multiple domains of clinical PTSD symptomology, in a subset of C57BL/6 mice (*n*=4/11 undergoing IFS; ∼36%) 8 weeks post-IFS index trauma, supporting the face validity and clinical relevance of the model (Fig. 2J).

### PTSD mice display loss of trabecular bone in femur and L3 vertebra 8 weeks post-IFS index trauma

**Fig. 3.**
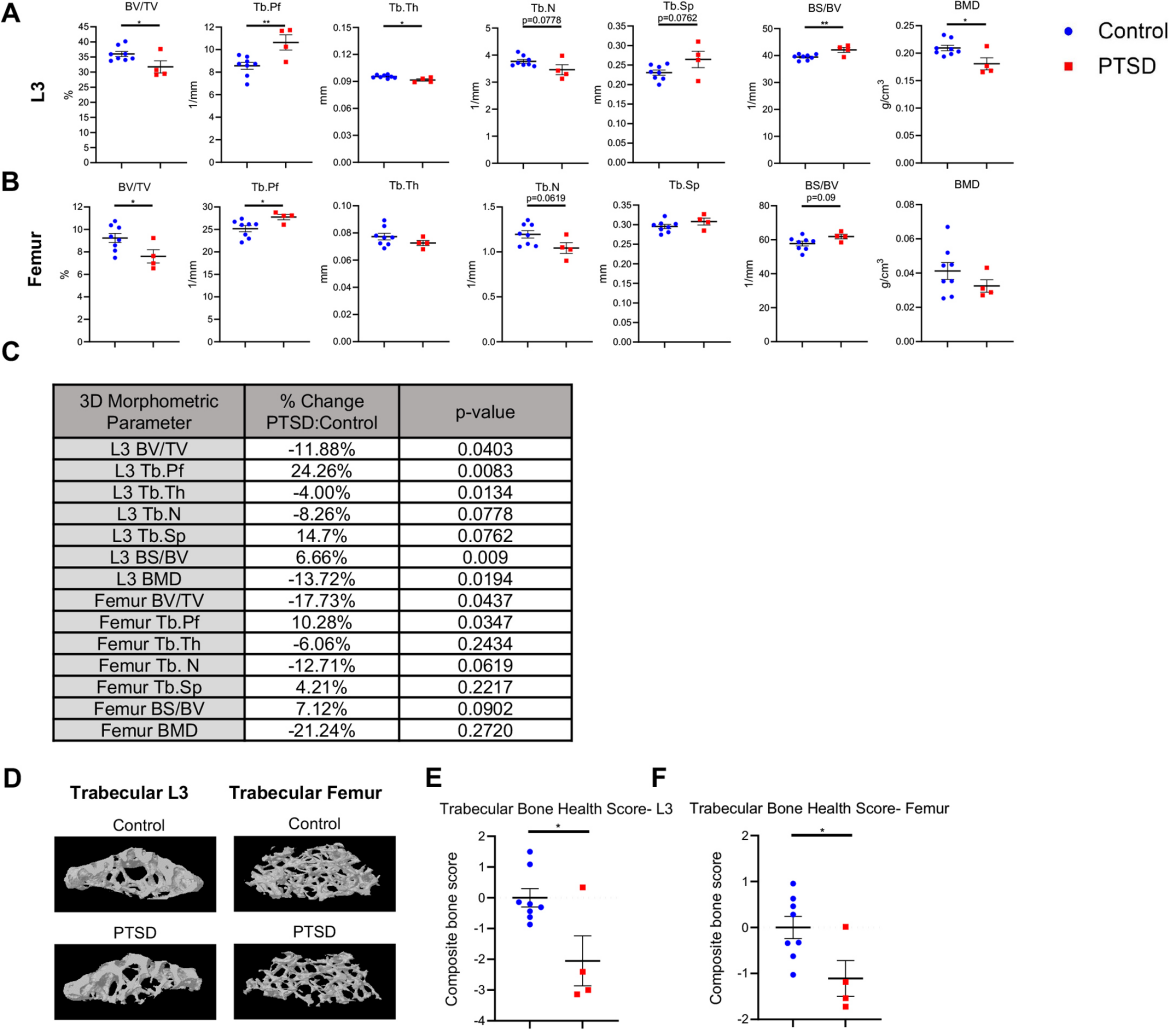
**PTSD mice display loss of trabecular bone in L3 vertebrae and femora.** (A,B) *Ex vivo* micro-CT scans were obtained on L3 vertebra (A) and femur (B), trabecular regions of interest were selected, and 3D morphometric parameters were calculated for PTSD and control mice at 8 weeks post-IFS index trauma. (C) The mean percentage change (PTSD versus control) is displayed. (D) 3D reconstruction of selected trabecular bone regions of interest of L3 and femur from representative control and PTSD mice. (E,F) An average composite score across trabecular bone morphometric parameters (trabecular bone health score) was calculated for control and PTSD mice for L3 vertebra (E) and femur (F). PTSD mice were characterized by a lower composite trabecular bone health score compared with control mice. Data are mean±s.e.m. Control, *n*=8; PTSD, *n*=4. Unpaired two-tailed Student's *t*-test (*P<0.05, **P<0.01).

To assess bone health in our IFS model, femora and L3 vertebrae from PTSD (*n*=4) and control (*n*=8) mice were examined using high-resolution *ex vivo* micro-CT at 8 weeks post-IFS index trauma (Fig. 3A-C). Using three-dimensional morphometric analyses, PTSD mice exhibited a significant decrease in bone volume/trabecular volume (BV/TV) in both femora (−17.7%, *P*=0.0437) and L3 vertebrae (−11.9%, *P*=0.0403) relative to control mice, demonstrating significant trabecular bone loss after the IFS paradigm. Trabecular pattern factor (Tb.Pf) was significantly increased in PTSD mice in femora (+10.3%, *P*=0.0347) and L3 (+24.3%, *P*=0.0083), indicating loss of trabecular architecture. Trabecular number (Tb.N) was decreased in PTSD mice for both femora (−12.7%, *P*=0.0619) and L3 (-8.3%, *P*=0.0778), while trabecular separation (Tb.Sp) was increased in both femora (+4.2%, *P*=0.2217) and L3 (+14.7%, *P*=0.0762). Trabecular thickness (Tb.Th) was significantly decreased in PTSD mice in L3 (−4%, *P*=0.0134), with a trend toward decrease in femora (−6.1%, *P*=0.2434). Bone surface to bone volume ratio (BS/BV) was significantly increased in L3 (6.66%, *P*=0.009), with a trend toward an increase in femora (7.12%, *P*=0.09). The BS/BV ratio is used as an indicator of the surface to volume relationship of the bone and is typically inversely correlated with Tb.Th. We observed a significant decrease in L3 bone mineral density (BMD) (−13.72%, *P*=0.0194) and a trend toward a decrease in femora density (−21.24%, *P*=0.2720). In humans, osteopenia is clinically defined by the World Health Organization as a density 10-25% below that of an average healthy adult. PTSD mice show a statistically significant average L3 BMD at 13.72% below control, and an average femoral BMD at 21.24% below control, suggesting a clinically relevant degree of osteopenia is induced in our IFS model. Three-dimensional reconstructions of L3 and femoral trabecular bone regions of interest illustrate the significant impact of PTSD on bone (Fig. 3D). To evaluate overall trabecular bone changes in PTSD mice relative to control mice, a composite score (i.e. trabecular bone health score) was calculated for each subject based on trabecular bone morphometric parameter calculations (i.e. BV/TV, Tb.Pf, Tb.Th, Tb.N, Tb.Sp and BS/BV) for both L3 and femur at 8 weeks post-IFS index trauma. PTSD mice exhibited significantly poorer trabecular bone health scores compared with control mice in L3 vertebrae (*P*=0.0143) and femora (*P*=0.0297) (Fig. 3E,F). Cortical bone was also assessed in femora and L3 vertebrae using three-dimensional morphometric analyses, with no significant changes observed in any morphometric parameter (Fig. S1). Taken together, these data demonstrate that IFS trauma leads to a cumulative loss of trabecular bone and changes in trabecular bone architecture in the femora and L3 vertebra of PTSD mice.

### PTSD mice exhibit lower bone health scores after IFS trauma

**Fig. 4.**
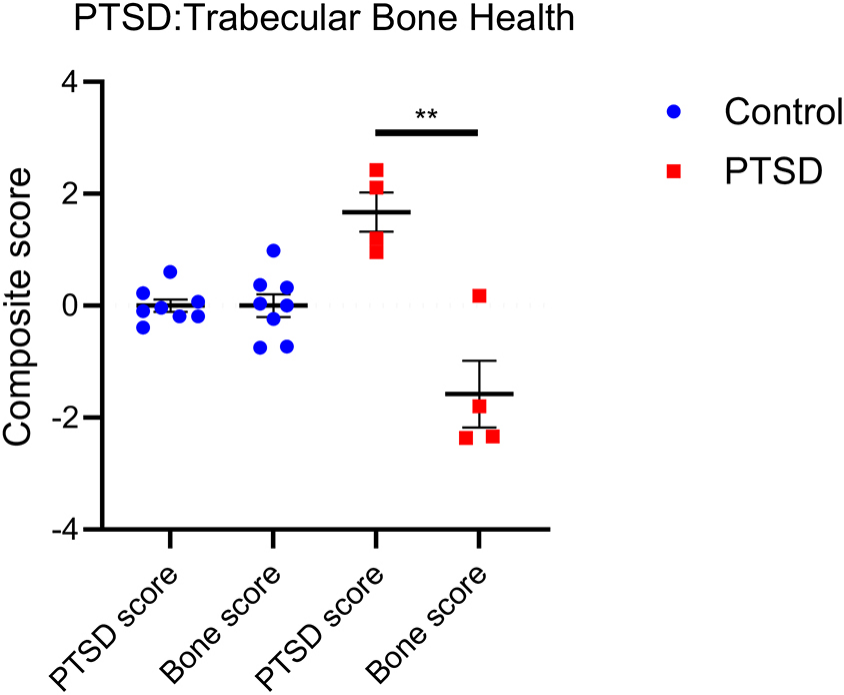
**PTSD score and trabecular bone health score demonstrate a negative relationship in female adult C57BL/6 mice.** Comparison of trabecular bone health score (averaged femur and L3 trabecular bone health scores) to PTSD score reveals a negative relationship between PTSD phenotype and trabecular bone health. Data are mean±s.e.m. Control, *n*=8; PTSD, *n*=4. Unpaired two-tailed Student's *t*-test (**P=0.0034).

To evaluate the relationship between PTSD and bone health in the IFS model 8 weeks post–IFS index trauma, composite behavioral scores (PTSD score) and composite trabecular bone scores (trabecular bone health score) were compared across control and PTSD mice. Comparison of PTSD score and bone score revealed a negative relationship between PTSD-like phenotype and bone health in PTSD mice (*P*=0.0034, Fig. 4). These data show that, in adult female C57BL/6 mice with a PTSD phenotype, trabecular bone health is compromised at 8 weeks post-IFS index trauma and provide evidence that the preclinical PTSD model developed herein may serve as a useful tool for studying impacts of PTSD on bone physiology.

### PTSD mice have lower trabecular bone health scores and altered bone material quality 15 weeks post-IFS index trauma

**Fig. 5.**
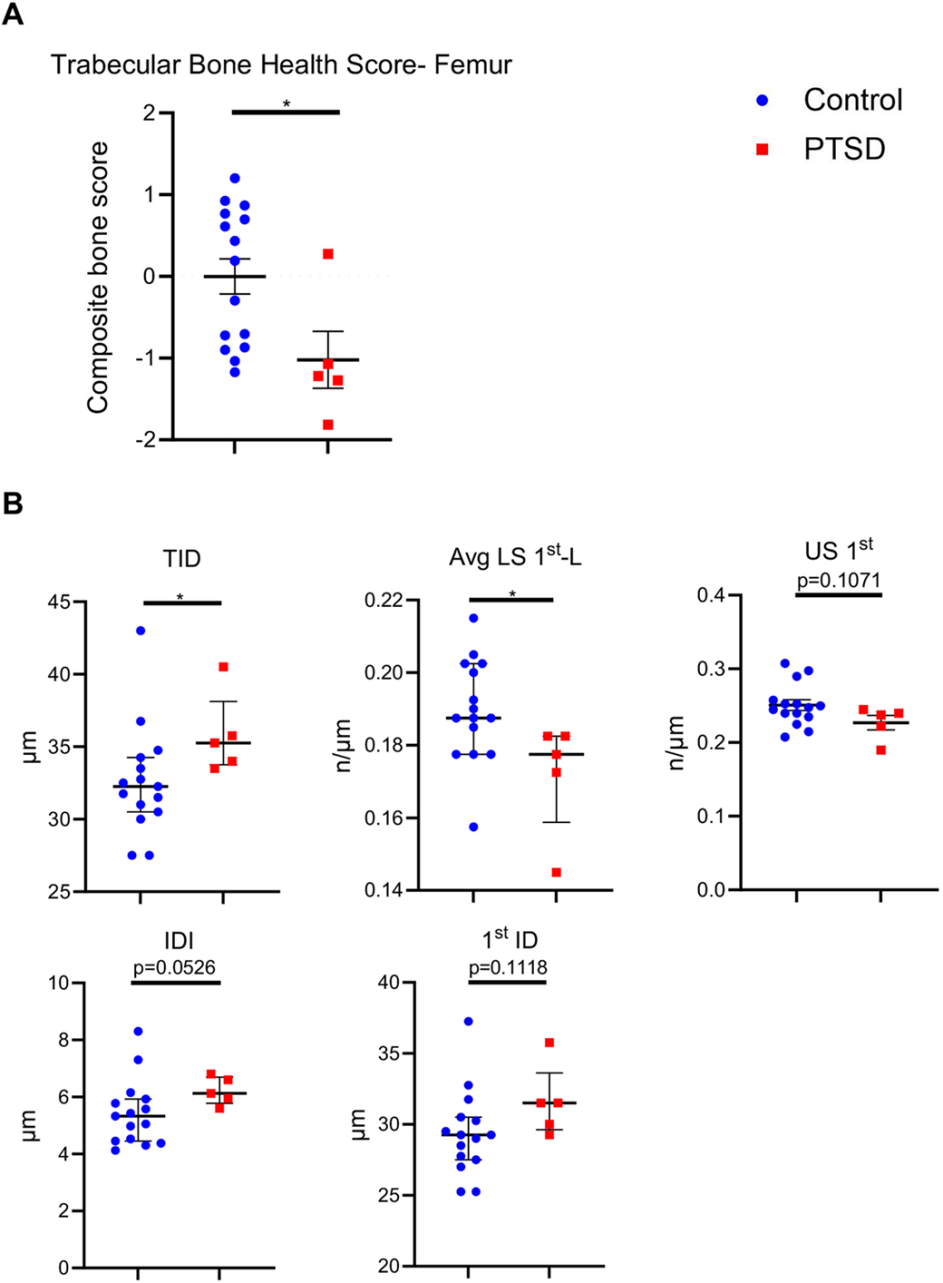
**PTSD leads to long-lasting alterations in trabecular bone and bone material quality.** (A) Trabecular bone health scores from femora were calculated for PTSD and control mice 15 weeks post-IFS index trauma. (B) To quantify bone material quality, cRPI calculations were performed on *ex vivo* femora from control and PTSD mice using a BioDent (2N, 10 cycles, 4 measurements/femur). Results suggest PTSD bones have altered bone mechanical properties and bone strength. Control, *n*=15; PTSD, *n*=5. Unpaired two-tailed Student's *t*-test (data shown as dot plots, mean±s.e.m.; A; B, US 1st) or Mann–Whitney *U* test (data shown as dot plots, median±interquartile range; B, 1st ID, TID, IDI and Avg LS 1st-L). *P<0.05.

We next wanted to examine the long-term effects of PTSD on bone. A second cohort of adult, female mice underwent the IFS paradigm and PTSD mice were classified at 8 weeks post-IFS index trauma, as above (*n*=5/15 undergoing IFS, ∼33%). Fifteen weeks post-IFS index trauma, three-dimensional morphometric analyses were conducted on PTSD (*n*=5) and control (*n*=15) femora, and trabecular bone health scores were calculated, as above. PTSD mice exhibited significantly poorer trabecular bone health compared with control mice (*P*=0.0278) (Fig. 5A). Cortical bone was also assessed in femora using three-dimensional morphometric analyses, with no significant changes observed in any morphometric parameter (Fig. S1). cRPI was performed on excised femora from this cohort at 15 weeks post-IFS index trauma to quantify bone material quality. PTSD femora exhibited a significant increase in total indentation distance (TID) (9.3%, *P*=0.0343) compared with control mice, which is inversely correlated to material toughness and suggests that PTSD reduces ability of bone matrix to resist crack initiation and propagation. A significant decrease in average loading slope (-5.33%, Avg LS 1st-L, *P*=0.0199) and a trend toward decreased unloading slope (US 1st, −8.81%, *P*=0.1071) suggest PTSD leads to decreased bone matrix stiffness and a decreased resistance to deformation. Trends toward increases in indentation distance increase (IDI, 15.02%, *P*=0.0526) and 1st indentation distance (1st ID, 7.69%, *P*=0.1118) in PTSD bones suggest these bones may be less resistant to fracture (Fig. 5B). Taken together, these data demonstrate that PTSD contributes to long-term alterations in femoral trabecular bone microarchitecture and impacts bone material quality.

### PTSD mice exhibit an increased percentage of empty lacunae, decreased cortical osteoid and increased trabecular TRAP staining in femora 15 weeks post-IFS index trauma

**Fig. 6.**
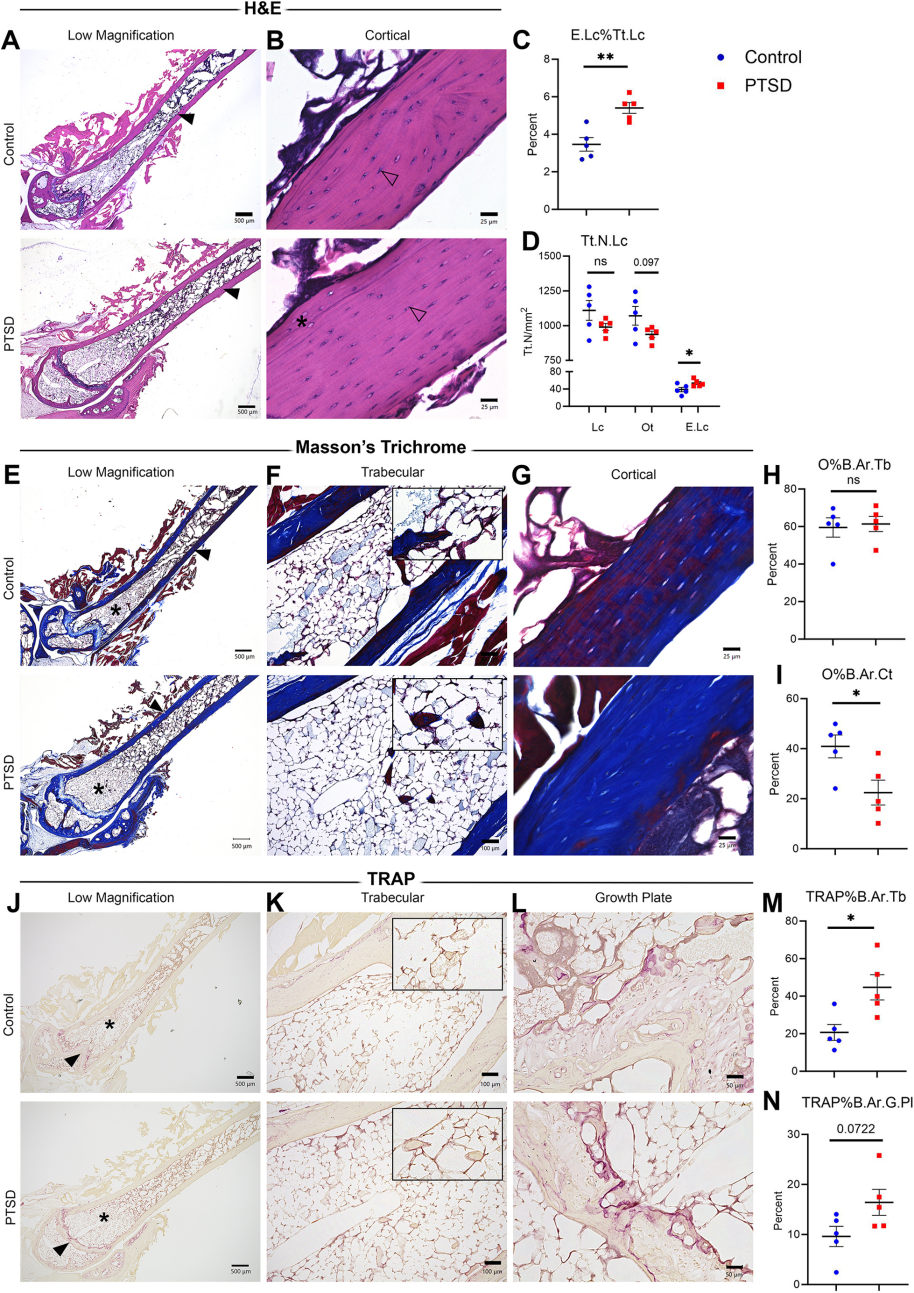
**Femora from PTSD mice exhibit an increased percentage of empty lacunae, decreased cortical osteoid and increased trabecular TRAP staining 15 weeks post-IFS index trauma.** (A-D) Total numbers per mm^2^ (Tt.N/mm^2^) of lacunae (L), osteocytes (O) and empty lacunae (E.Lc) were quantified in Hematoxylin and Eosin-stained femur sections from a subset of control and PTSD mice (15 sections/group) (open arrowheads indicate osteocyte within lacunae, asterisk indicates empty lacunae). (E-I) Total area of osteoid in trabecular (O%B.Ar.Tb) and cortical (O%B.Ar.Ct) bone was quantified in Masson's trichrome-stained femur sections from control and PTSD mice (14 or 15 sections/group). (J-N) Total area of TRAP was quantified in trabecular (TRAP%B.Ar.Tb) and growth plate (TRAP%B.Ar.G.Pl) bone areas of TRAP-stained femur sections from control and PTSD mice (14 or 15 sections/group). Data are mean±s.e.m. Control, *n*=5; PTSD, *n*=5. Unpaired two-tailed Student's *t*-test (*P<0.05, **P<0.01). Black arrowheads and asterisks in A, E and J indicate regions shown in representative higher magnification images (B, G, K and L). (F,K) Insets in show representative tissue analyzed.

To investigate the impacts of PTSD on the cellular compartments of bone, histological analyses were conducted on excised femoral bone from PTSD and control mice 15 weeks post-IFS index trauma. Total area of bone analyzed for histomorphometry was not statistically different in PTSD versus control mice (Fig. S2). Total (Tt) and empty lacunae (Lc) were quantified in PTSD versus Control femoral bone (Fig. 6A,B). There was no significant change in the mean number of total lacunae (Tt.N.Lc) (990.4 versus 1110 Lc/mm^2^, *P*=0.1512), but a significant increase in the total number of empty lacunae (E.Lc) (+38.1%, 53.6 versus 38.8 Lc/mm^2^, *P*=0.0445) and therefore a significant increase in the percentage of empty to total lacunae (E.Lc%Tt.Lc) in PTSD versus control bone (5.4 vs 3.5%, *P*=0.0030) (Fig. 6C,D). Masson's Trichrome staining (Fig. 6E-G) showed a significant decrease in the total area of osteoid in the cortical bone (O%B.Ar.Ct) of PTSD mice relative to control mice (−45.2%, *P*=0.0259) (Fig. 6I). No change in the total area of osteoid in trabecular bone (O%B.Ar.Tb) was observed in PTSD versus control mice (*P*=0.7799, Fig. 6H). TRAP staining (Fig. 6J-L) revealed an increase in total area of TRAP in trabecular bone (TRAP%B.Ar.Tb) of PTSD mice compared with control mice (+116.2%, *P*=0.0164) (Fig. 6M). A trend toward an increase in the total area of TRAP near the growth plate (TRAP%B.Ar.G.Pl) was also observed in PTSD versus control mice (+70.9%, *P*=0.0722) (Fig. 6N). There was no significant difference in mean thickness of the femoral distal growth plate (G.Pl.Th) between PTSD and control mice (130.2 versus 135.9 µm/measurement, *P*=0.1411). No change in total area of TRAP in cortical bone (TRAP%B.Ar.Ct) was observed in PTSD versus control mice (*P*=0.8413, Fig. S3). Overall, these data demonstrate that PTSD bone is negatively impacted at the cellular level and points to a potential dysregulation in bone turnover leading to net bone resorption.

### Mice with a PTSD-like phenotype exhibit sustained systemic inflammation 15 weeks post-IFS index trauma

**Fig. 7.**
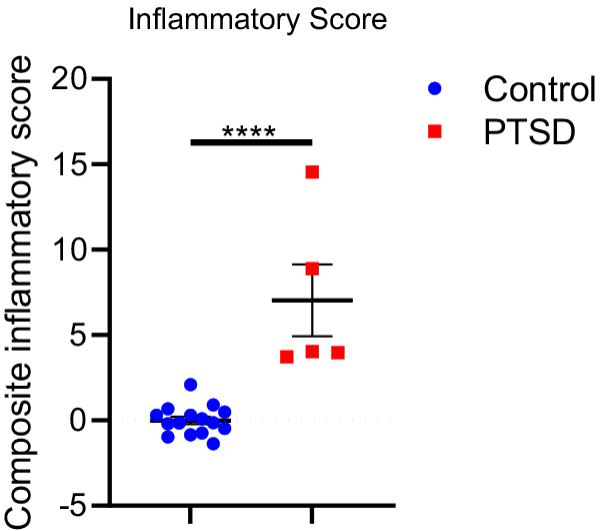
**PTSD mice exhibit a higher inflammatory score compared with control mice.** An average composite score across all spleen cell-produced inflammatory mediators (inflammatory score; see Table 1) was calculated for control and PTSD mice 15 weeks after IFS index trauma. PTSD mice were characterized by a higher inflammatory score compared with control mice. Data are mean±s.e.m. Control, *n*=15; PTSD, *n*=5. Unpaired two-tailed Student's *t*-test (****P<0.0001).

Given the reported roles of inflammation in PTSD and bone pathophysiology, we examined systemic inflammation in PTSD mice. Spleens were harvested from PTSD (*n*=5) and control (*n*=15) mice 15 weeks post-IFS index trauma. Spleen cell-produced immune mediators, including Th1 cell-, Th2 cell-, Th17 cell- and inflammatory macrophage-associated factors, were quantitated and compared between groups. Compared with splenocytes from control mice, splenocytes from PTSD mice produced significantly increased levels of IFNγ (*P*<0.0001), IL2 (*P*<0.0001), IL6 (*P*<0.0001), TNFα (*P*<0.0001), RANTES (*P*<0.0001), MIG (*P*=0.0015), MIP1α (*P*<0.0001), MIP1β (*P*<0.0001), G-CSF (*P*=0.0003) and GM–CSF (*P*<0.0001) (Table 1). To assess the inflammatory phenotype in each subject, a composite score (i.e. inflammatory score) was calculated for each subject based on levels of inflammatory mediators. PTSD mice exhibited an increased inflammatory score compared with control mice (*P*<0.0001), indicating that PTSD mice exhibit a sustained and persistent inflammatory phenotype post-IFS index trauma (Fig. 7). These data support the construct validity of this model and suggest that increased inflammation may contribute to PTSD-related bone loss.

**Table 1.**
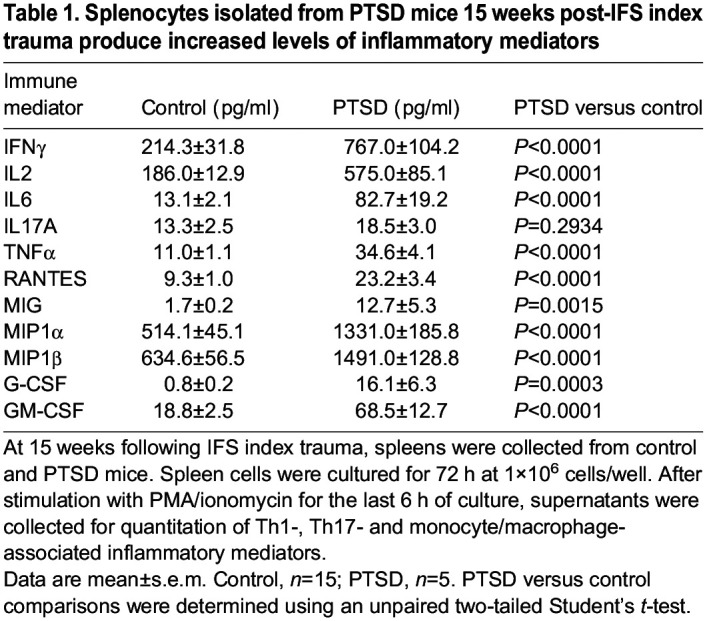
Splenocytes isolated from PTSD mice 15 weeks post-IFS index trauma produce increased levels of inflammatory mediators

## DISCUSSION

Although there is much discussion on what defines an ideal animal model of PTSD in the fields of behavioral science and neurobiology (Richter-Levin et al., 2019; Flandreau and Toth, 2018; Deslauriers et al., 2018), there are several generally accepted criteria: face validity (recapitulates symptomology), construct validity (models changes in neurobiology and/or physiology), predictive validity (models clinical response to treatment) and inter-individual variability in response (Yehuda et al., 1993; Richter-Levin et al., 2019; Verbitsky et al., 2020). Previous studies show that variations of the electric shock paradigm induce behavioral changes, PFC/amygdala dysfunction, alterations in hippocampal morphology and increased inflammation analogous to that observed in PTSD patients (Aspesi and Pinna, 2019; Cheng et al., 2018; Milad and Quirk, 2002; Golub et al., 2011). However, few studies report on PTSD–induced changes in bone phenotype. One earlier study showed that adolescent mice exposed to psychosocial stress displayed decreased femur/tibia length and increased growth plate thickness, suggesting that PTSD may negatively impact bone growth (Foertsch et al., 2017; Haffner-Luntzer et al., 2019). Chronic psychosocial stress-induced PTSD has been shown to impact immune cell recruitment and, consequently, fracture healing, in adult male mice, providing further evidence for PTSD as a disruptor of bone health and healing (Haffner-Luntzer et al., 2019). Additionally, a recent study examining the impact of social isolation on bone loss in mice revealed significant effects in male mice (Mountain et al., 2023). However, despite these findings, no studies to date have developed a murine model of PTSD-induced bone loss that closely mimics the clinical features observed in human patients. Therefore, the present study aimed to address this gap.

In the current study, we present a murine model of PTSD in adult female mice exhibiting high face and construct validity, as well as inter-individual variability in response. We began our studies in female mice as women are twice as likely to develop PTSD compared with men (Haskell et al., 2010). Clinically, osteoporosis and low bone mass have been shown to be more prevalent among women than men in the USA (Sarafrazi et al., 2021). Although a few previous studies have suggested that female mice may more readily display anxiety-related behaviors after IFS (freezing, avoidance, etc.) (Kokras and Dalla, 2014; Shansky, 2015; Deslauriers et al., 2018), robust modeling of PTSD in female mice is lacking. Therefore, we developed the current IFS paradigm in adult female mice (Fig. 1). The initial IFS session serves as the index trauma (Fig. 1). Eight weeks after IFS index trauma, a subset of mice, ‘PTSD mice’, exhibit persistent freezing behavior in the trauma-associated context (IFS arena) in response to the trauma-specific (tone) cue (Fig. 2). Although re-living the traumatic event cannot be quantifiably measured in small animals, trauma-specific freezing has been accepted as a reasonable index of clinical symptom criteria B. Importantly, given that only a subset of these IFS mice (PTSD, ∼33-36%) exhibit persistent freezing in response to trauma, this paradigm preserves the individual variance in response to trauma observed in the PTSD patient population, supporting the clinical relevance and etiological validity of our model (Cohen et al., 2006). As freezing behavior is observed in PTSD mice 8 weeks after initial trauma, this model also meets clinical symptom criteria F and surpasses behavioral endpoints used in many previous animal studies, typically concluding 10 days to 1 month after index trauma. This is a distinguishing characteristic of the presented model and underscores its potential for the study of long-term pathophysiology in bone and other organ systems in the context of PTSD. In addition to trauma-associated freezing in the IFS context, PTSD mice display cognitive deficits (criteria D, sociability test), increased reactivity (criteria E, locomotor test) and freezing in response to the trauma-specific tone in a novel context, 8 weeks post-IFS index trauma. These phenotypic changes are reflected in the higher composite ‘PTSD Score’, compared with control mice. This model is based on a combination of social stress and IFS to induce PTSD; however, it is important to note that single housing alone impacts behavior in mice and likely contributes to the anxiety-related behavior observed in PTSD mice. Together, the behavioral assessments provide evidence that the presented model displays high face validity, mimics the variability in response to trauma observed in the PTSD patient population and exhibits long–term changes in phenotype that may serve as a useful tool for the study of comorbidities (e.g. bone disorders) in the context of PTSD.

Multiple clinical studies show that PTSD patients exhibit reduced bone mass and increased fracture risk (Azuma et al., 2015; Huang et al., 2018; El-Gabalawy et al., 2018; Hain et al., 2011). The model presented herein produces significant, long-lasting negative impacts on bone, as demonstrated by micro-CT, cRPI and histological analyses (Figs 3-6). Interestingly, 15 weeks after the IFS index event, femora of PTSD mice showed an increased percentage of empty lacunae versus total lacunae compared with control mice, although total lacunae density was unchanged, indicating a disruption in osteocyte biology in PTSD mice. Masson's trichrome staining showed decreased osteoid formation in cortical, but not trabecular, bone in PTSD versus control mice. These changes indicate aberrant bone remodeling dynamics in the cortical compartment at the cellular level. Intriguingly, 3D morphometric analyses via micro-CT did not show changes in cortical bone parameters, suggesting a need for a longer-term study of PTSD impacts on cortical bone at the whole animal level. Concomitantly, PTSD femora exhibited increased TRAP staining in trabecular bone and a trend towards increased TRAP staining near the growth plate compared with control femora. No change in TRAP staining was observed in diaphyseal cortical bone, suggesting that increased TRAP activity is localized to the trabecular compartment. Together, these data indicate that PTSD may impact the net balance of osteoblasts:osteoclasts in mature bone and lead to a dysregulated state of bone turnover at the cellular level that persists long after initial trauma. Although the specific drivers of this imbalance will be investigated in subsequent studies, bone loss has been associated with fewer osteocytes (Qiu et al., 2003; Mullender et al., 1996), and there have been reports of correlations between bone remodeling indices and the density of empty osteocyte lacunae (Hedgecock et al., 2007; Zarrinkalam et al., 2012). Regardless of the cause, loss of osteocytes has been associated with increased osteoclast recruitment, osteoclast activity, bone resorption and bone remodeling (Verborgt et al., 2000; Noble et al., 1997; Cardoso et al., 2009; Emerton et al., 2010), and this is supported by our data.

Additionally, PTSD mice exhibited increased splenic levels of Th1 cell-, Th2 cell- and macrophage–associated cytokines/mediators, as reflected by a higher inflammatory score, which further supports the construct validity of the model. This observed increase in inflammation may represent a continued dysregulation of immune balance that impacts systemic physiology and signaling long term, and, importantly, reflects clinical findings. PTSD patients are characterized by increased systemic levels of inflammatory cytokines, including IFNγ, TNFα and IL17A, as well as increased levels of inflammatory immune cells, including Th1 and Th17 cells (Hori and Kim, 2019; Wang et al., 2017; Zhou et al., 2014; Von Kanel et al., 2007; Wang et al., 2016; Guo et al., 2012). These factors have been implicated in driving bone pathophysiology through direct and indirect regulation of osteoclast differentiation and osteoblast apoptosis (Eastell et al., 2016; Pietschmann et al., 2016). IL1, IL6 and TNFα have been shown to stimulate osteoclast function, while simultaneously inhibiting osteoblast function. TNFα directly promotes osteoclast development and also decreases osteoprotegerin (OPG) release by osteoblasts, thus indirectly promoting osteoclast activity (Pietschmann et al., 2016). These studies suggest that there may be intersecting inflammatory mechanisms driving the development and progression of both PTSD and bone pathophysiology. The current study evaluated global changes in spleen cell-produced inflammatory mediators as part of model characterization. Future studies will delineate individual inflammatory cell populations and factors in PTSD mice to investigate the spatial and temporal contributions of specific inflammatory cell subsets to the observed bony phenotype.

Taken together, the current study presents a murine model of PTSD that exhibits clinically relevant changes in behavior, inflammation and bone physiology. These data support a robust link between psychological stress and bone, and mirror changes observed in PTSD patients. Although this model focuses on females, there are also likely impacts of this PTSD paradigm on bone physiology in adult male mice, and this will be an important area of future study that may reveal both intersecting and unique sex-dependent impacts of PTSD on bone health. With regard to mechanisms, there are several signaling pathways involved in stress and chronic stress signaling that intersect with bone pathophysiology, including global increases in catecholamines, changes in IGF, serotonin and RANKL signaling, immune activation, and dysregulated glucocorticoid and stress hormone signaling (reviewed by Kelly et al., 2019). We propose that our model could be used in future studies to elucidate the roles of each of these key signaling pathways in the context of PTSD–induced bone loss, to identify unique markers for PTSD-related skeletal comorbidity and to provide new insight into mechanisms at the intersection of PTSD and bone health.

## MATERIALS AND METHODS

### Animal models

10-week-old, female B6.SJL-Ptprc^a^Pepc^b^/BoyJ (C57BL/6) mice were obtained from Jackson Laboratories (Bar Harbor, Maine, USA) and acclimated to the facility for 1 week before study initiation (i.e. beginning of social isolation). Mice for each cohort arrived in the same shipment and were randomized into study groups. Mice were maintained in the Ralph H. Johnson VA Health Care System (RHJ VAHCS, Charleston, SC, USA) Animal Research Facility. Research was conducted in accordance with the US Public Health Service Policy on Humane Care and Use of Laboratory Animals and RHJ VAHCS Institutional Animal Care and Use Committee (IACUC) guidelines.

### Behavioral studies

#### Social isolation

Beginning 1 week prior to inescapable foot shock (IFS), IFS group mice were singly housed and remained singly housed for the duration of the study. Control mice were group housed for the duration of the study.

#### Inescapable foot shock

On Day 0, IFS mice were placed singly into inescapable 17 cm by 17 cm arenas within sound-attenuated chambers and allowed to habituate for 1 min before delivery of five 1 s, 1 mA foot shocks over a 5 min trial. Each shock was preceded by and co-terminated with a 20 sec, 80 dB, 9 kHz tone cue. Control mice were placed singly into arenas and allowed to habituate for 1 min before delivery of five 20 s, 80 dB, 9 kHz tone cues over a 5 min trial. Control mice did not receive any foot shocks over the trial. Freezing behavior (velocity, cm/s) was recorded with an IR-camera at 25 frames/s and EthoVision XT v13 software (Noldus Information Technology; Wagenigen, the Netherlands).

#### Trauma reminder

One week post-IFS index trauma, IFS mice were placed singly into the same arena context and allowed to habituate for 3 min before delivery of a single, 1 s, 1 mA foot shock preceded by and co-terminating with a 20 s, 80 dB, 9 kHz tone cue. Control mice were placed singly into the same arenas and allowed to habituate for 3 min before delivery of a single 20 s, 80 dB, 9 kHz tone. Control mice did not receive any foot shocks over the trial. Freezing behavior (velocity, cm/s) was recorded following the tone-shock or tone delivery with an IR camera at 25 frames/s and EthoVision XT v13 software.

#### Trauma reminder tests in IFS context

Four and 8 weeks after initial IFS, IFS and control mice were placed singly into the same arena context and allowed to habituate for 3 min before delivery of a 20 s, 80 dB, 9 kHz tone cue. Mice were allowed to explore freely for 3 min after the tone-shock or tone delivery. Freezing behavior (velocity, cm/s) was recorded following the tone-shock or tone delivery with an IR camera at 25 frames/s and EthoVision XT v13 software. PTSD mice were defined as those who exhibited freezing behavior after the tone cue in this context at 8 weeks post-IFS index trauma and were selected based on the following criterion: velocity_PTSD_ >1.5 standard deviations below mean velocity_Control_.

#### Sociability test

Eight weeks after initial IFS, IFS and control mice were placed singly into a three-chambered sociability arena to evaluate sociability. The arena consisted of three equally sized chambers separated by clear plexiglass walls, each containing an opening through which the mice could freely move. A cage with plexiglass bars was placed into each of the left and right chambers. Each test consisted of two trials. For the habituation trial, each mouse was placed into the center chamber and allowed to freely explore for 5 min. The mouse was then briefly removed from the arena. An unfamiliar, age- and sex-matched conspecific was then placed into the cage in the left chamber. For the sociability trial, the test mouse was placed back into the center chamber and allowed to freely explore for 5 min. Overall locomotion and latency to enter the zone directly surrounding the conspecific subject was recorded using an overhead camera at 25 frames/s and EthoVision XT v13 software.

#### Locomotor test

Eight weeks after initial IFS, changes in locomotor activity were evaluated using the CatWalk XT system. For this test, each mouse was placed at the start of an enclosed, dark corridor and allowed to freely cross an illuminated platform to the goal box, under which the home cage was housed, for a total of three compliant runs. Compliancy was based on an overall run time of 0.5-5 s per run and a threshold of <60% variability in velocity within runs. Body velocity (cm/s) across the platform was recorded by an under-mounted, high-speed color camera and CatWalk XT software.

#### Trauma reminder test in OFA context

Eight weeks after initial IFS, IFS and control mice were singly placed into 40 cm by 40 cm plexiglass open field arenas and allowed to explore freely. Mice were allowed to habituate for 3 min before delivery of a 20 s, 80 dB, 9 kHz tone cue. Mice were allowed to explore freely for 3 min after the tone delivery. Freezing behavior (velocity, cm/s) was recorded during the tone delivery with an overhead camera at 25 frames/s and EthoVision XT v13 software.

### Bone analyses

#### Micro-computed tomography

High-resolution (9 µm on-a-side cubic voxels; 180°; 0.5° rotation; 2 frame average; 0.5 mm Al filter; 50 kV x-ray source voltage; 500 µA x-ray source current) micro-computed tomography (micro-CT) images were obtained using the Bruker SkyScan 1176 (Bruker Corporation, Billerica, MA, USA) from excised right femora and spines of female PTSD and control 20- to 21-week-old mice 8 weeks post-IFS index trauma or from excised femora of 27- to 28-week-old female mice 15 weeks post-IFS index trauma. The femora were wrapped in saline-soaked gauze and stored at −20°C. Before analysis, they were thawed at 4°C. Two-dimensional transaxial and three-dimensional images were reconstructed for qualitative and quantitative analyses using the Bruker software suite (reconstruction parameters: smoothing 2; ring artifacts reduction 10; beam hardening 35%). The region of interest (ROI) for femoral analysis was selected relative to the distal femoral growth plate. For trabecular femoral bone analysis, a 1.3 mm ROI offset 0.35 mm proximally from the distal femoral growth plate was selected. For cortical femoral bone analysis, a 2.6 mm ROI offset 2.6 mm proximally from the distal femoral growth plate was selected. Automated thresholding was applied to segregate trabecular from cortical bone, following the Automated Trabecular and Cortical Bone Selection Method Note provided by Bruker microCT. For vertebral analysis, the L3 vertebra was cropped and oriented, and a 0.87 mm ROI surrounding the midpoint of the vertebral body, with reference to the caudal and cranial growth plates, was selected. Trabecular bone was segmented using a manually drawn interpolated ROI, while cortical bone was separated using a subtractive ROI. Three-dimensional morphometric analyses were conducted on each sample to evaluate trabecular and cortical bone parameters. Bone mineral density (BMD) and tissue mineral density (TMD) were calculated based on scanned phantoms of known densities. The bone parameters were analyzed using the Custom Analysis program (CTAn, Skyscan, Bruker, Belgium).

#### BioDent

Force versus displacement data were obtained from excised femora of female 27- to 28-week-old PTSD and control mice 15 weeks post-IFS index trauma using the BioDent system (ActiveLife Scientific, Santa Barbara, CA, USA). The femora were wrapped in saline-soaked gauze and stored at −20°C. Before analysis, they were thawed at 4°C. The femora were cleaned of soft tissue and placed on the bed of the system while submerged in phosphate-buffered saline. A reference probe was positioned 6000 µm proximal to the patella against the anterior midshaft. Using the BP3 probe, a 2 N load was applied for 10 cycles. Four measurements were taken per femur, with a 750 µm proximal distance adjustment after each measurement. The reference force ranged between 260 and 310 g, and the touchdown distance ranged between 50 and 250 µm for each measurement.

#### Histology

Hindlimbs were excised from 27- to 28-week-old female mice 15 weeks post-IFS index trauma in both PTSD and control groups. Hindlimbs were fixed in 4% paraformaldehyde for 24 h and stored in 0.05% sodium azide. Decalcification was performed using Newcomer's Supply Decalcifying Solution, EDTA/sucrose (Newcomer's Supply, Middleton, WI, USA) with gentle agitation for 72 h. After decalcification, hindlimbs were embedded in paraffin wax and longitudinally sectioned at 5 µm. The sections were then deparaffinized, dehydrated and stained with Hematoxylin and Eosin (Vector Laboratories, Newark, CA, USA) or Masson's trichrome (IHC World, Ellicot City, MD, USA) according to manufacturer protocols. For tartrate-resistant acid phosphatase (TRAP) staining, the sections were deparaffinized and dehydrated. Freshly prepared TRAP buffer containing 0.1 M acetate buffer, 0.3 M sodium tartrate, Triton-X 100, 100 mg/ml Napthol AS-MX phosphate (Sigma N-5000, Sigma-Aldrich, St. Louis, MO, USA) and deionized H_2_O was prewarmed in a 37°C water bath. Slides were submerged in the buffer for 14-18 min, rinsed in PBS and counterstained with Hematoxylin for 2 min. After counterstaining, the slides were rinsed three times with distilled H_2_O and cover-slipped using Permount (Electron Microscopy Sciences, Hatfield, PA, USA).

#### Histomorphometry

Tissue sections from hindlimbs of PTSD and control groups (*n*=5/group), collected 15 weeks post-IFS index trauma, were examined using a Keyence BZ-X810 microscope and BX-X800 Analyzer software (Keyence Corporation of America, Itasca, IL, USA). Bright-field microscopy was used to analyze non-overlapping fields of view within the femoral bone. Areas of analysis included both mineralized bone and osteoid, excluded bone marrow (Dempster et al., 2013), and were anatomically similar between control and PTSD sections. Total area of bone analyzed was not statistically different between control and PTSD mice (Fig. S2) and all data are normalized to total bone area (mm^2^). High magnification (200×, 400×) images were acquired using *z* stack imaging (5 µm range, 0.3 µm pitch). For cortical bone histological analyses, regions of interest correlated with regions of interest analyzed by cRPI. Total numbers of osteocytes (Tt.N.Ot) and lacunae (Tt.N.Lc) were quantified at 400× magnification, with four fields of view assessed per section (15 sections/group). The ratio of empty to total lacunae (E.Lc%Tt.Lc) was then calculated. Masson's trichrome staining was conducted to evaluate the total area of osteoid in trabecular (O%B.Ar.Tb, one field of view/section, 100×) and cortical (O%B.Ar.Ct, three fields of view/section, 200×) bone in 14 or 15 sections/group. Hue-based thresholding and batch analysis techniques were used to accurately quantify the osteoid relative to mature bone. The total area of TRAP stain was quantified in trabecular bone (TRAP%B.Ar.Tb, one field of view/section, 100×), cortical bone (TRAP%B.Ar.Ct, two fields of view/section, diaphyseal, 200×) and near the growth plate (TRAP%B.Ar.G.Pl, two fields of view, 200×) in 14 or 15 sections/group. Hue–based thresholding and batch analysis were used to accurately quantify the TRAP-stained area.

### Immune analyses

#### Spleen processing

Spleens were harvested from PTSD and control mice 15 weeks following IFS trauma. The spleens were homogenized using a glass homogenizer, and the resulting cell suspension was passed through a 70 µm strainer (BD Falcon, San Jose, CA, USA). The cells were then rinsed with Hank's Balanced Salt Solution (HBSS, Life Technologies, Grand Island, NY, USA), and red blood cells were lysed by adding ammonium-chloride-potassium (ACK) Lysing Buffer (Lonza, Walkersville, MD, USA) for 3 min. Splenocytes were washed twice with HBSS, and the cell number was determined by counting the cells excluding Trypan Blue using a hemocytometer.

#### Spleen cell culture

Splenocytes were cultured for 72 h at 37°C in fresh media at a density of 1×10^6^ cells/well in 12-well anti–CD3-coated tissue culture plates with 30 IU mouse IL-2 (R&D Systems, Minneapolis, MN). During the last 6 h of culture, the cells were stimulated with 1X cell stimulation cocktail (Thermo Fisher Scientific, Waltham, MA, USA). After stimulation, supernatants were collected for quantitation of inflammatory cytokines using the Mouse Cytokine Array/Chemokine Array 31 Plex Assay (MD31, Eve Technologies, Calgary, Alberta, Canada).

### Statistical analyses

Statistical analyses were conducted using Microsoft Excel (Microsoft, Redmond, WA, USA) or GraphPad Prism software (GraphPad Software, La Jolla, CA, USA). To evaluate PTSD-like phenotype in each subject, a composite score (Song et al., 2013) was calculated for each behavioral assessment (trauma reminder tests in IFS and OFA contexts, sociability test, locomotor test) 8 weeks post-IFS index trauma, as follows: [(x - µ_C_)/s_C_], where x=subject value, µ_C_=control mean and s_C_=control standard deviation. Averaged composite scores of all behavioral assessments (i.e. PTSD score) were then calculated for each subject. In some cases, data (i.e. velocity) were inverted to retain directionality in which a more positive score equates to more PTSD-like phenotype. To evaluate trabecular bone health in each subject, a composite score was calculated for trabecular bone parameters (BV/TV, Tb.Pf, Tb.Th, Tb.N, Tb.Sp, BS/BV) 8 weeks or 15 weeks after IFS index trauma, using the same formula as the PTSD score. Averaged composite scores across all trabecular bone parameters were then calculated to obtain the trabecular bone health score for each subject. When necessary, specific parameter data (i.e. Tb.Pf, Tb.Sp, BS/BV) was inverted to retain directionality in which a more negative score equates to poorer bone outcomes. Similarly, a composite score was calculated for each cortical bone parameter (CV, VIP, Md.V, Ct.Th, FD, CS/CV and TMD) using the same formula as above. The averaged composite scores of all cortical bone parameters yielded the cortical bone health score for each subject. When necessary, specific parameter data (i.e. CS/CV) was inverted to retain directionality in which a more negative score equates to poorer bone outcomes. To evaluate systemic inflammation, a composite score was calculated for each spleen cell-produced immune mediator (IFNγ, IL2, IL6, IL17A, TNFα, RANTES, MIG, MIP1α, MIP1β, G-CSF and GM-CSF) following the same procedure as above. The averaged composite scores of all immune mediators yielded the inflammatory score for each subject. Equal weighting was given to all parameters in all score calculations. Data are presented as mean±s.e.m. in dot plots. Normally distributed datasets were analyzed using an unpaired two-tailed Student's *t-*test. Nonnormally distributed datasets were analyzed using the Mann–Whitney *U*-test. *P*≤0.05 was considered statistically significant (*P≤0.05, **P≤0.01, ***P≤0.001 and ****P≤0.0001) indicate the level of statistical significance.

## Supplementary Material

10.1242/dmm.050044_sup1 Supplementary information
