# Supplementary material for: Inescapable foot shock induces a PTSD-like phenotype and negatively impacts adult murine bone
Source: Dis Model Mech. 2024 Jan 12;17(1):dmm050044. doi: 10.1242/dmm.050044 (PMC10820809; doi:10.1242/dmm.050044)
Supplement: Supplementary information [file dmm-17-050044-s1.pdf]

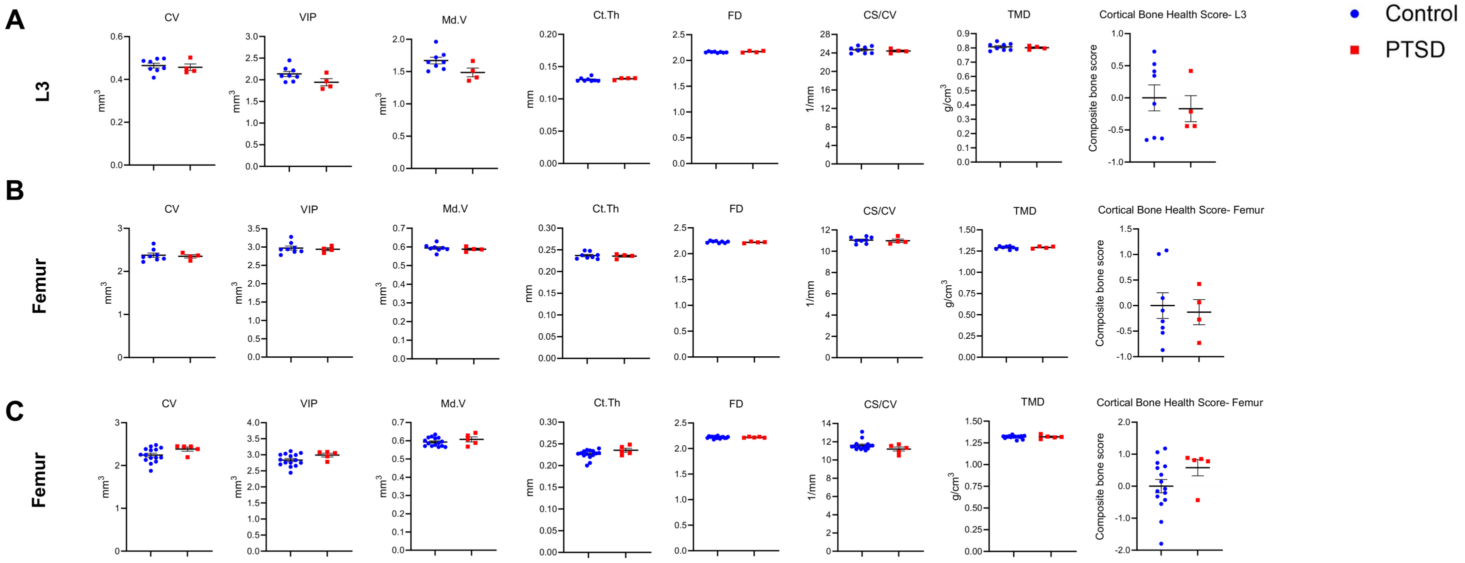

Fig. S1. Micro-CT does not demonstrate significant changes in cortical bone 3D morphometrics in PTSD mice. *Ex vivo* micro-CT scans were obtained of L3 vertebrae (A) and femora (B) at 8 weeks post-IFS index trauma and femora (C) at 15 weeks post-IFS index trauma. Cortical regions were selected, and 3D morphometric parameters were calculated (CV, cortical volume; VIP, volume inside periosteum; Md.V, medullary volume; Ct.Th, cortical thickness; FD, fractal dimension; CS/CV, cortical surface/cortical volume; TMD, tissue mineral density). An average composite score across all cortical bone morphometric analyses was calculated for Control and PTSD mice for L3 vertebrae and femora (Cortical Bone Health Score). Data shown as mean ± s.e.m. Control n=8; PTSD n=4 (A, B); Control n=15; PTSD n=5 (C). Student's two-tailed t test, unpaired.

Disease Models & Mechanisms • Supplementary information

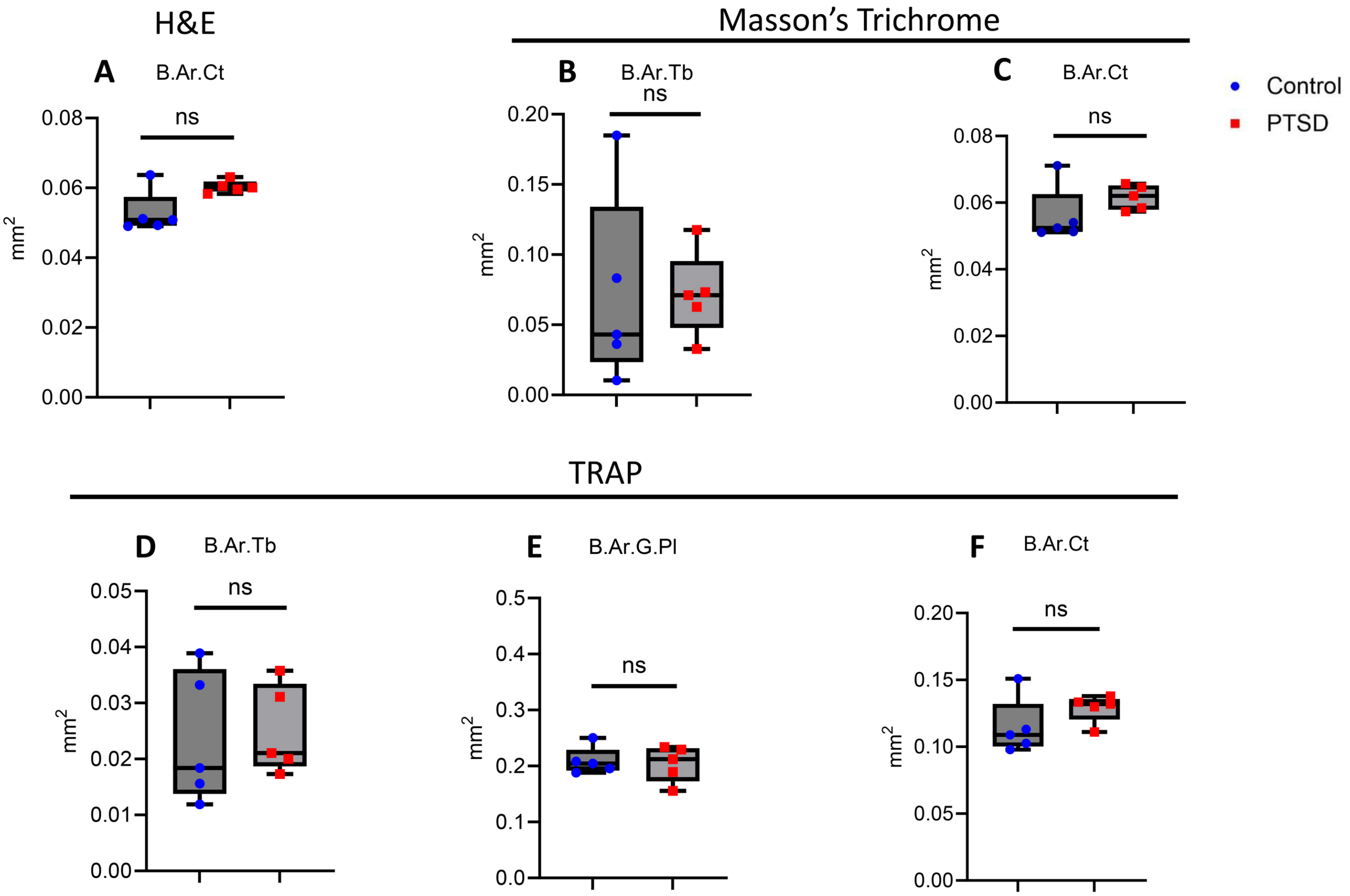

Fig. S2. Area of bone analyzed for histomorphometry was not statistically different in PTSD versus Control mice. Area (mm^2) of cortical bone (B.Ar.Ct) analyzed in H&E-stained femur sections in Control and PTSD mice (15 sections/group) (A). Area (mm^2) of trabecular (B.Ar.Tb) and cortical (B.Ar.Ct) bone analyzed in Masson's Trichrome-stained femur sections in Control and PTSD mice (14-15 sections/group) (B, C). Area (mm^2) of trabecular (B.Ar.Tb), growth plate (B.Ar.G.Pl), and cortical (B.Ar.Ct) bone analyzed in TRAP-stained femur sections in Control and PTSD mice (14-15 sections/group) (D-F). Data shown as box and whisker plots to illustrate maximum and minimum values, with individual data points superimposed. Control n=5; PTSD n=5. Student's two-tailed t test, unpaired (B, D, E, F), Mann-Whitney *U* test, (A, C).

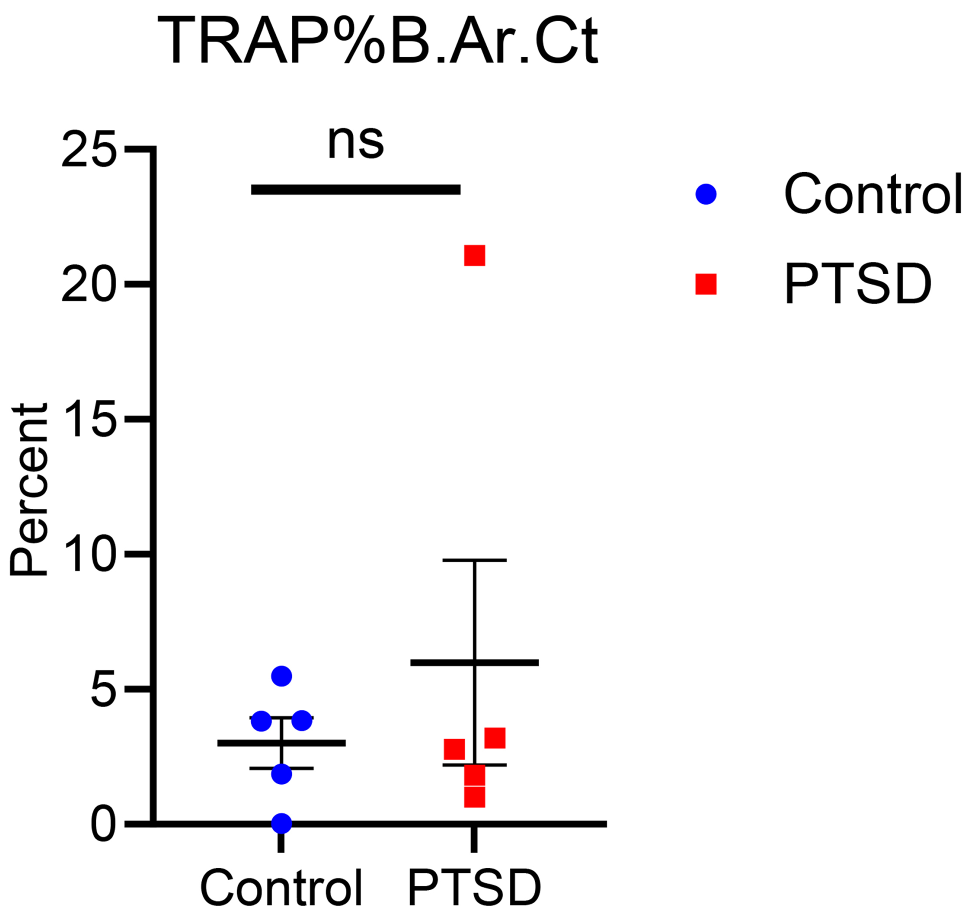

Fig. S3. Femora from PTSD mice exhibit no change in cortical TRAP staining 15 weeks post-IFS index trauma. Total area of TRAP was quantified in cortical (TRAP%B.Ar.Ct) bone areas of TRAP-stained femur sections from Control and PTSD mice (14-15 sections/group). Data shown as dot plot, median ± interquartile range. Control n=5; PTSD n=5. Mann-Whitney *U* test.

Disease Models & Mechanisms • Supplementary information
